# Depicting Precise Temperature and Duration of Vernalization and Inhibiting Early Bolting and Flowering of *Angelica sinensis* by Freezing Storage

**DOI:** 10.3389/fpls.2022.853444

**Published:** 2022-05-19

**Authors:** Xiaoxia Liu, Mimi Luo, Mengfei Li, Jianhe Wei

**Affiliations:** ^1^State Key Laboratory of Aridland Crop Science, College of Life Science and Technology, Gansu Agricultural University, Lanzhou, China; ^2^Institute of Medicinal Plant Development, Chinese Academy of Medical Sciences and Peking Union Medical College, Beijing, China

**Keywords:** *Angelica sinensis* (Oliv.) Diels, vernalization, early bolting and flowering, freezing storage, physiological characteristics

## Abstract

*Angelica sinensis* is a perennial rhizomatous herb that is widely used for the treatment of cardio-cerebrovascular diseases, which largely rely on metabolites, such as alkylphthalides, polysaccharides, and ferulic acid. This plant must experience low-temperature vernalization and long-day conditions for the occurrence of early bolting and flowering (EBF) that reduces yield and quality of fleshy root. In current commercial planting, the EBF of more than 40% is mainly attributed to the completion of vernalization of seedlings during overwinter storage. While effects of storage temperatures [vernalization temperature (0–10°C) and freezing temperature (−2 to −12°C)] and seedling sizes on the EBF have been observed in previous studies, the precise vernalization temperature and duration for different size seedlings, the effective freezing storage to avoid vernalization of seedlings, and physiological characteristics have not been systematically investigated. Here, the EBF rate, the anatomical structure of shoot apical meristem (SAM), and physiological characteristics of different size seedlings at different storage temperatures (0, 3, 5, −3, and −5°C) and durations (14–125 d) are reported. The vernalization duration of seedlings was predicated from 57 to 85 d with temperatures ranging from 0 to 5°C based on the linearization regression analysis *via* Matrix Laboratory software. The EBF can be effectively inhibited by freezing storage. The anatomical structure of SAM, levels of primary metabolites (soluble sugar, starch, amino acid, and protein), and endogenous hormones (GA_3_, IAA, and ABA), exhibited a dynamical change in the seedlings at different storage temperatures. These findings will provide useful information for predicting the vernalization of seedlings and inhibiting the EBF in large-scale commercial cultivation.

## Introduction

*Angelica sinensis* (Oliv.) Diels (family Umbelliferae), common names as Danggui, Dong quai, Tang kuei, and Chinese angelica, is a perennial herbaceous species (McGuffin et al., 2000). It prefers growing in cool-moist conditions at an altitude of 2,200 to 3,000 m and is widely cultivated in western parts of China, including Gansu, Qinghai, Sichuan, and Yunnan (Zhang and Cheng, 1989; Zhang et al., 2012; Xu et al., 2020). The roots have been used as a traditional Chinese medicine for nourishing and activating the blood, regulating female menstrual disorders and relieving pains, relaxing bowels, etc., over 2,000 years (Upton, 2003; Committee for the Pharmacopoeia of PR China, 2015; Wei et al., 2016). In recent years, the roots are also been applied in the treatment of cardio-cerebrovascular diseases as well as agents of anti-inflammatory and antioxidant (Upton, 2003; Wang and Ou-Yang, 2005; Chao et al., 2010; Wang et al., 2015), which largely rely on the bioactive components, including alkylphthalides, polysaccharides, ferulic acid, and essential oils (Ma et al., 2015; Wei et al., 2016; Li et al., 2022).

Currently, the cultivated area of *A. sinensis* is more than 43,500 ha due to increasing demand for clinical application (Zhang et al., 2012; Huang and Jin, 2018). In the commercial large-scale cultivation, seeds are sown in summer and germinated seedlings are collected in autumn to be overwintered indoors; in the spring, stored seedlings are planted out for vegetative growth and either harvested in autumn of this second year for fleshy roots or kept in the field until mid-summer of the third year for seed collection (Supplementary Figure 1; Huang and Jin, 2018; Li et al., 2021). However, the 2-year-old plants occur up to 40% EBF, which makes the roots lignified along with the reduction in yield and quality (Supplementary Figure 2); furthermore, the lignified roots are useless in medicinal agents due to little accumulation of the bioactive components (Zhang et al., 2018; Li et al., 2020a,b, 2022).

To inhibit the occurrence of the EBF, several efforts being made include selecting the cultivars with a lower rate of EBF (Huang and Jin, 2018), controlling the seedling sizes (Lin et al., 2007), investigating the type of vernalization and photoperiod (Wang, 1977), storing the seedlings below freezing temperatures (Wang, 1977, 1979; Jia et al., 2017, 2018), and avoiding the plants grown in the long-day conditions (Yao, 2005). Specifically, Huang and Jin (2018) reported that the EBF rate of the green stem cultivar (Mingui 2) was lower than the purple stem cultivar (Mingui 1), while the Mingui 1 occupied over 95% cultivation area due to better growth characteristics. Lin et al. (2007) demonstrated that there was a positive relationship of EBF rates with seedling sizes, with the EBF rates of 73, 47, 25, and 6% under the root shoulder diameter ≥0.66, 0.56–0.65, 0.46–0.55, and ≤0.45 cm, respectively. Wang (1977) found that *A. sinensis* is a “low-temperature and long-day” plant and the transition from vegetative growth to flowering must satisfy with vernalization (0–5°C) and long-day conditions (>12 h daylight). Based on the type of vernalization and photoperiod, the vernalization could be avoided after the seedlings are stored at −2 to −12°C (Wang, 1979; Jia et al., 2018); the long-day conditions could be also avoided under 40, 60, and 80% sunshade with the EBF rates 16.3, 12.3, and 5.3%, respectively (Yao, 2005). In the practical large-scale cultivation to inhibit or reduce the EBF, avoiding the vernalization of seedlings stored in a smaller freezing room is far more feasible than shading the long-day conditions of plants grown in a larger sunshade field (Wang, 1977).

Although effective temperature range and duration for vernalization of *A. sinensis* seedlings have been reported in previous literature, their results for vernalization temperature (VT) and duration (VD) are inconsistent (Wang, 1977; Li, 1979; Zhang and Huang, 1998). Specifically, Wang (1977) found that the effective temperature for vernalization ranged from 0 to 5°C; Li (1979) reported that more than 80°C accumulated temperature was required for flowering with the seedlings exposed to 5–10°C for a certain duration; and Zhang and Huang (1998) reported that the vernalization could complete at 4°C for 170 d. Furthermore, changes in physiological characteristics, such as the levels of soluble sugar, protein, malonic dialdehyde, and nitrate reductase, were observed when the seedlings were stored at different vernalization and freezing temperatures (Zhang and Huang, 1998; Chen et al., 2014). The abovementioned literature indicate that the studies on the precise VT and VD for different seedlings (in other words the most effective conditions to induce EBF), the effective freezing storage to avoid vernalization of seedlings, and physiological characteristics during storage are still limited, which lead to the problem of the EBF having not been resolved so far. In this study, the effects of storage temperatures [vernalization temperature (VT, 0–5°C) and freezing temperature (FT, −3 to −5°C)] and durations (14–125 d) on the rotting or chilling damages, EBF, anatomical structure of SAM, levels of primary metabolites, and endogenous hormones in different size seedlings were systematically investigated by the methods of paraffin section, spectrometry, and HPLC.

## Materials and Methods

### Plant Materials

The mature seeds of 3-year-old *A. sinensis* (cultivar Mingui 1) were sown (3,020 m a.s.l.; 36°59′ 41″N, 102°4′ 29″E) on June 26, 2020. After 108 d germination and growth *in situ*, the seedlings were dug out on October 11, 2020 (Supplementary Figure 3). After leaving in outdoor for about 10 d to evaporate water content to 70–75%, the seedlings were stored at different temperatures (0, 3, 5, −3, and −5°C). After certain days (specifically, 0°C for 14, 21, 25, 30, 45, 50, 60, 75, 81, 90, 104, and 125 d; 3 and 5°C for 14, 21, 30, 45, 60, 75, and 90 d; and −3 and −5°C for 25, 50, 81, 104, and 125 d) (Table 1), the seedlings were taken out and divided into three grades [large (0.5–0.6 cm), medium (0.4–0.5 cm), and small sizes (0.3–0.4 cm)] based on the diameter of root shoulder measured by a digital caliper, to observe the anatomical structure of SAM, to measure physiological characteristics (e.g., root activity, contents of soluble sugar, starch, protein, and amino acid as well as GA_3_, IAA, and ABA), and to investigate the EBF rate with the seedlings planted in a pot (13 cm × 9 cm) with soil (coconut coir: peat: fermented cow dung: pearlite = 3:3:2:2) and grown at the greenhouse (20°C, 12-h daylight long-day condition).

**TABLE 1:** Duration of seedlings stored at different temperatures.

Temperature (^°^C)	Duration (d)											
0	14	21	25	30	45	50	60	75	81	90	104	125
3	14	21	/	30	45	/	60	75	/	90	/	/
5	14	21	/	30	45	/	60	75	/	90	/	/
-3	/	/	25	/	/	50	/	/	81	/	104	125
-5	/	/	25	/	/	50	/	/	81	/	104	125

*The “/” indicates seedlings were not collected.*

### Survey of Germination and Growth Characteristics

The rotting rate (RR) and freezing injury rate (FIR) of seedlings were immediately surveyed with vernalization (0, 3, and 5°C) at 21, 30, and 45 d and freezing temperatures (−3 and −5°C) at 70, 104, and 125 d; the germination rate (GR) and EBF rate were surveyed after the seedlings grown at 30 and 90 d, respectively. The specific calculations are as follows:

RR (%) = (number of rotten roots of seedlings / n) × 100%, (note: *n* = 800–1,000)
FIR (%) = (number of frozen injury roots of seedlings/n) × 100%, (note: *n* = 800–1,000)
GR (%) = (number of germination roots of seedlings n) × 100%, (note: *n* = 54)
EBF rate (%) = (number of EBF plants/n) × 100%, (note: *n* = germinated and survived plants, *ca.* 30–50)

### Observation of Shoot Apical Meristem

The anatomical structure of shoot apical meristem (SAM) was observed using a paraffin section method with slight modifications (Li et al., 2016, 2020c). Briefly, the roots were first rinsed, and the root shoulders containing the SAM with leaf primordia (0.5 cm) were cut off and immersed into FAA fixative solution (70% ethanol: formaldehyde: glacial acetic acid = 90:5:5, v/v) at 4°C for 12 h. Second, the FAA-fixed samples were washed three times with 70% ethanol at 22°C for 10 min and then sequentially dehydrated in ethanol 30% (4 h), 50% (4 h), 70% (3 h), 85% (3 h), 95% (2.5 h), 100% (2 h), and 100% (1.5 h). Third, the dehydrated samples were sequentially transparentized in the mixture (2:1, 1:1, 1:2, and 0:1 v/v) of ethanol and dimethylbenzene for 2 h, respectively. Fourth, the transparentized samples were sequentially immersed in the mixture (1:1 and 2:1 v/v) of dimethylbenzene and paraffin at 56°C for 12 h, immersed in paraffin three times at 58°C for 12 h, and then embedded in 2 cm paraffin cubes. Finally, the anatomical structure of SAM was observed by a reset one inverted microscope (Revolve RVL-100-G, ECHO, CA, United States) after the embedded samples were sliced (7 μm) with a rotary microtome (KD-2258, Cody, Jinhua, China) and stained with safranin O fast green FCF (S8020, Solarbio, Beijing, China; F8130, Solarbio, Beijing, China).

### Measurement of Physiological Characteristics

#### Measurement of Root Activity

Root activity was measured using a triphenyl tetrazolium chloride (TTC) method (Li and Zhang, 2016). Briefly, the roots were rinsed and cut into small pieces (5 mm). The pieces (0.5 g) were put into 10 mL tubes, and then TTC (3 mL, 0.4% w/v) and phosphate buffer (3 mL, pH 7) were added in sequence. After oscillation and reaction in dark at 37°C for 40 min, sulfuric acid (2 mL, 1 mol/L) was added to stop the reaction. The samples were dried with absorbent paper, grind into homogenate in ethyl acetate (5 mL), and centrifuged at 5,000 r/min at 4°C for 5 min, and then, the supernatant was increased to 9 mL with ethyl acetate. Absorbance was measured at 485 nm, root activity was evaluated based on μg of TTC, and the standard curve of TTC was attached in Supplementary Table 1.

### Measurement of Soluble Sugar, Starch, Protein, and Amino Acid Contents

#### Preparation for Extracts

The extracts were prepared according to a published protocol with slight modifications (Yang et al., 2016). Briefly, the freshly collected roots were first air-dried in a ventilated room; the roots were finely ground, and the powder (2.0 g) was soaked in ethanol (15 mL, 10% v/v) and agitated in a shaker with 120 r/min at 24°C for 8 h; the homogenate was centrifuged at 6,000 r/min at 4°C for 10 min and re-extracted twice more. The supernatant was increased to 50 mL with ethanol (10% v/v) and then kept at 4°C for measurement.

### Measurement of Soluble Sugar Content

Soluble sugar content was measured using a phenol–sulfuric acid method (Dubois et al., 1956). Extracts (10 μL) were added to phenol reagent (1 mL, 9% v/v), and sulfuric acid (3 mL) was added after oscillation which then reacted at 22°C for 30 min. Absorbance was measured at 485 nm, the soluble sugar content was evaluated based on mg of sucrose, and the standard curve of sucrose was attached in Supplementary Table 1.

### Measurement of Starch Content

Starch content was measured using an anthrone colorimetry method (Cao et al., 2018). Briefly, of the soluble sugar residues, ddH_2_O (15 mL) was added and boiled for 15 min; perchloric acid (2 mL, 9.2 mol/L) was added to the mixture and then boiled for 15 min; the homogenate was centrifuged at 5,000 r/min at 4°C for 10 min. The supernatant was increased to 25 mL with ddH_2_O. Extracts (20 μL) were added into ddH_2_O (2 mL) and anthrone–sulfuric acid (6 mL) and then boiled for 7 min. Absorbance was measured at 640 nm, starch content was evaluated based on mg of soluble starch, and the standard curve of soluble starch was attached in Supplementary Table 1.

### Measurement of Protein Content

Protein content was measured using a Coomassie brilliant blue colorimetric method (Bradford, 1976). Briefly, extracts (70 μL) were added into Coomassie brilliant blue G-250 protein reagent (5 mL) and then reacted at 22°C for 2 min. Absorbance was measured at 595 nm, protein content was evaluated based on mg of bovine serum albumin, and the standard curve of bovine serum albumin was attached in Supplementary Table 1.

### Measurement of Amino Acid Content

Amino acid content was measured using a ninhydrin coloration method (Wang, 2006). Briefly, extracts (450 μL) were sequentially added into ammonia-free distilled water (1 mL), ninhydrin hydrate (3 mL), and ascorbic acid (0.5 mL); the mixture was put into an 80°C water bath to react for 20 min; after cooling to temperature, the mixture was increased to 20 mL with ammonia-free distilled water. Absorbance was measured at 570 nm, amino acid content was evaluated based on mg of leucine, and the standard curve of leucine was attached in Supplementary Table 1.

### Quantification of GA_3_, IAA, and ABA Contents

The extracts were prepared according to a published protocol with slight modifications (Pan et al., 2010). Briefly, the freshly collected root shoulders containing the SAM with leaf primordia (0.5 g) were first extracted with methanol (7 mL, 80% v/v) at 4°C for 8 h; the homogenate was centrifuged at 8,000 r/min at 4°C for 10 min and re-extracted twice more; the supernatant was concentrated at 40°C by a rotary evaporator, and then, the concentrate was adjusted to pH 8.0 with Na_2_HPO_4_ (0.4 mol/L); second, the petroleum ether was added to the concentrate with the volume 1:1 (v/v) to decolor and repeated twice more; third, the residual petroleum ether in the concentrate was removed at 40°C by a rotary evaporator, and then, the concentrate was adjusted to pH 2.8 with citric acid (0.4 mol/L); fourth, the decolored concentrate was extracted thrice with ethyl acetate (1:1, v/v) and then concentrated at 40°C by a rotary evaporator; finally, the concentrated residue was increased to 5 mL with methanol and then kept at −20°C for quantification.

The extracts of endogenous hormones were first filtered with a durapore membrane (0.22 μm), and the samples (5 μL) were injected and quantified at 254 nm using an HPLC (Agilent 1260 Infinity II, CA, United States) with Symmetry C_18_ column (250 mm × 4.6 mm, 5.0 μm, CA, United States) at a column temperature of 25°C and flow rate of 1.0 mL/min. Methanol (A)–phosphoric acid (0.1% v/v, B) was the mobile phase with gradient elution: 0–1 min 10–45% (A), 1–3 min 45–55% (A), 3–5 min 55–65% (A), 5–8 min 65–75% (A), 8–12 min 75–10% (A), and 12–14 min 10–10% (A). The contents of GA_3_, IAA, and ABA were quantified based on standard references (Supplementary Figure 4), and the standard curves were attached in Supplementary Table 2.

### Statistical Analysis

All the measurements were performed using three biological replicates. Statistical analysis was performed *via* ANOVA and Duncan’s multiple comparison tests, and SPSS 22.0 was the software package used with *P* < 0.05 as the basis for statistical differences. The Matrix Laboratory (MATLAB) software was used to program the linearization regression analysis for the relationship of EBF rate with vernalization temperature (VT), vernalization duration (VD), and seedlings classification (SC), with the number “1, 2, and 3” representing SC “small, medium, and large size,” respectively.

## Results

### Effect of Storage Temperature and Duration on Rotting and Freezing Injury

**FIGURE 1:**
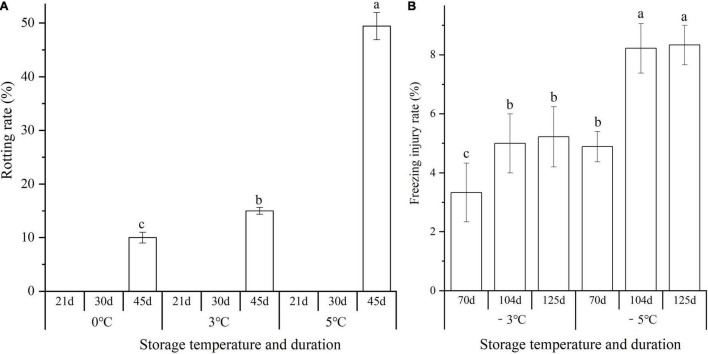
Changes in the rotting rate (RR) and freezing injury rate (FIR) of seedlings at different storage temperatures and durations. Panels **(A,B)** represent the RR and FIR, respectively. Different lowercase letters represent a significant difference (*P* < 0.05) in the average value of different seedlings (large, medium, and small sizes) among different temperatures and durations.

Significant differences in the rotting rate (RR) and freezing injury rate (FIR) of seedlings (including large, medium, and small sizes) were observed at different storage temperatures and durations. The rotting occurred after the seedlings were stored above 0°C for 45 d with the RR of 10.0, 15.0, and 49.4 at 0, 3, and 5°C, respectively (Figure 1A). The FIR at −3 and −5°C increased by 1.6- and 1.7-fold from 70 to 125 d, and the FIR at −5°C exhibited a 1.6, 3.2, and 3.1% higher than that of the −3°C at 70, 104, and 125 d, respectively (Figure 1B).

### Effect of Storage Temperature and Duration on Germination

**FIGURE 2:**
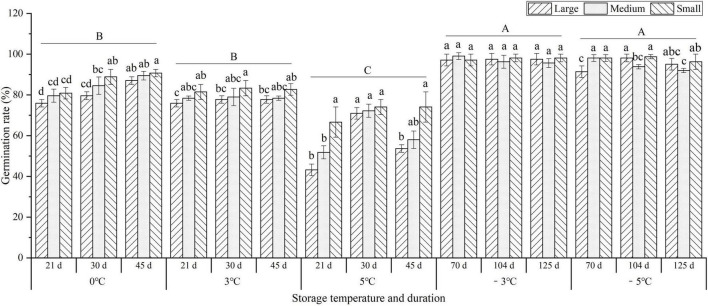
Changes in the germination rate (GR) of seedlings at different storage temperatures and durations. Different uppercase letters represent a significant difference (*P* < 0.05) in the average value of different seedlings and storage durations among different temperatures. Different lowercase letters represent a significant difference between the size treatments at different temperatures.

To investigate the effects of storage temperature and duration on the germination of seedlings, the germination rate (GR) was observed at 0, 3, 5, −3, and −5°C. There was a 1.2-, 1.2-, and 1.6-fold decrease in the average GR for the different seedlings (large, medium, and small sizes) at 0, 3, and 5°C compared to −3°C, respectively, but there was no significant difference between −3°C (97.4%) and −5°C (95.7%). In addition, the GR of the small size was higher than that of the large and medium sizes at 0, 3, and 5°C, but no obvious difference for large, medium, and small sizes was observed at −3 and −5°C (Figure 2).

### Effect of Storage Temperature and Duration on Early Bolting and Flowering

**FIGURE 3:**
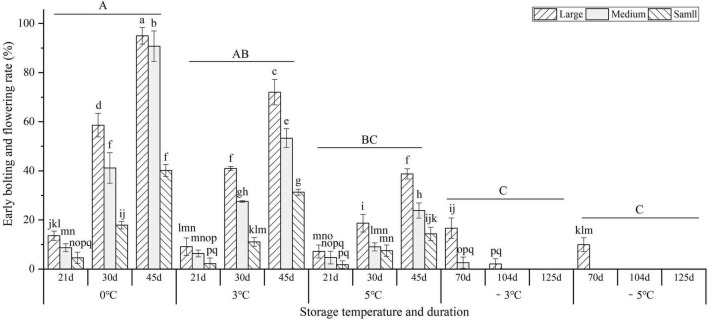
Changes in the early bolting and flowering (EBF) rates of seedlings at different storage temperatures and durations. Different uppercase letters represent a significant difference (*P* < 0.05) in the average value of different seedlings and storage durations among different temperatures. Different lowercase letters represent a significant difference between the size treatments and storage durations among different temperatures.

As shown in Figure 3, storage temperature and duration had significant effects on the early bolting and flowering (EBF) rate. At the vernalization temperatures (0, 3, and 5°C), the EBF rates significantly increased with prolonged storage from 21 to 45 d, but significantly decreased with temperature increase; the highest EBF rate occurred at 0°C for 45 d with large, medium, and small sizes reaching 95.0, 90.8, and 40.2%, respectively. At the freezing temperatures (−3 and −5°C) for 70 d, the highest EBF rates for large sizes were 16.7 and 9.9%, but there was no EBF occurring for the medium and small sizes, which indicates that freezing storage (below −3°C) can effectively inhibit the EBF. In addition, the EBF rates were observed to be highest in the large size, followed by the medium and small sizes.

### Predicting the Precise Vernalization Temperature and Duration

Based on the linearization regression analysis on the relationship of EBF rate with VT, VD, and seedlings classification (SC), the regression model was obtained as the following equation:


$$EBFrate\left(\%\right)=1.85\times VD+0.19\times VT^{3}-2.04\times VT^{2}+12.86\times SC-43.17$$

where *VD* (d), *VT* (0, 3, and 5^°^C), and *SC* (1, 2, and 3) with the number “1, 2, and 3” represent “small, medium, and large sizes,” respectively.

**FIGURE 4:**
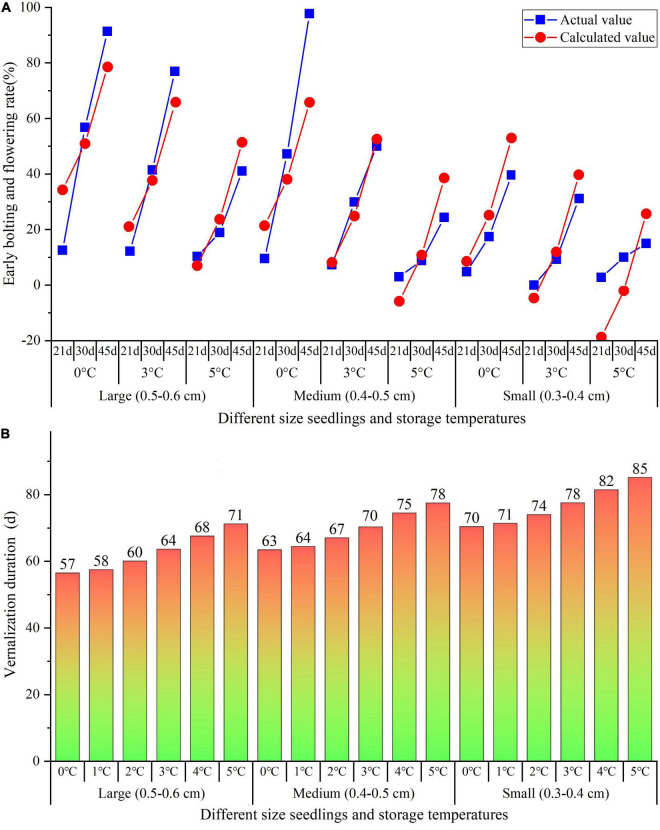
Linearization regression analysis on the relationship of EBF rate with VT, VD, and SC **(A)**, as well as speculated the precise VT and VD of different size seedlings **(B)**. VT, vernalization temperature; VD, vernalization duration; and SC, seedlings classification.

In comparison of the actual EBF rate in this study with the calculated EBF rate by the equation, there was no difference between the actual and calculated curves that were close together (Figure 4A). Based on the above equation, the precise VD to complete the vernalization for flowering (EBF rate = 100%) was predicted with the VT ranging from 0 to 5^°^C and SC from 1 to 3 being available. As shown in Figure 4B, the VD ranges from 57 to 71 d with VT from 0 to 5^°^C for the large size, 63–78 d for the medium size, and 70–85 d for the small size.

### Effect of Storage Temperature and Duration on Anatomical Structure of Shoot Apical Meristem

**FIGURE 5:**
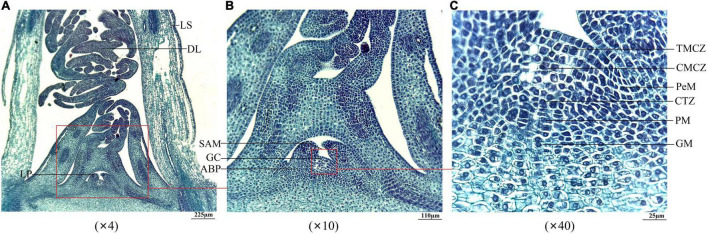
Longitudinal anatomical structure of the SAM of seedlings of *Angelica sinensis*. Panels **(A–C)** represent the structure at 4×, 10×, and 40× magnification of the large size at the −5°C for 25 d, respectively. LS, leaf sheath; DL, delicate leaf; LP, leaf primordium; GC, growth cone; ABP, axillary bud primordium; TMCZ, tunica mother cell zone; CMCZ, central mother cell zone; PeM, peripheral meristem; CTZ, cambium-like transition zone; PM, pith meristem; GM, ground meristem.

Since *A. sinensis* is a triennial plant in commercial cultivation, the vernalization of seedlings stored at the overwintering stage will confer to the ability to EBF in the second year (Supplementary Figures 1–3). To investigate the effects of storage temperature and duration on the SAM, first, the anatomical structures of the SAM were observed. As shown in Figure 5, the observed samples contained leaf sheath (LS), delicate leaves (DL), leaf primordium (LP), and SAM (Figure 5A); the SAM was composed of the growth cone (GC) and axillary bud primordium (ABP) (Figure 5B); the SAM that could be specifically distinguished at 40× magnification included tunica mother cell zone (TMCZ), central mother cell zone (CMCZ), peripheral meristem (PeM), cambium-like transition zone (CTZ), pith meristem (PM), and ground meristem (GM) (Figure 5C).

**FIGURE 6:**
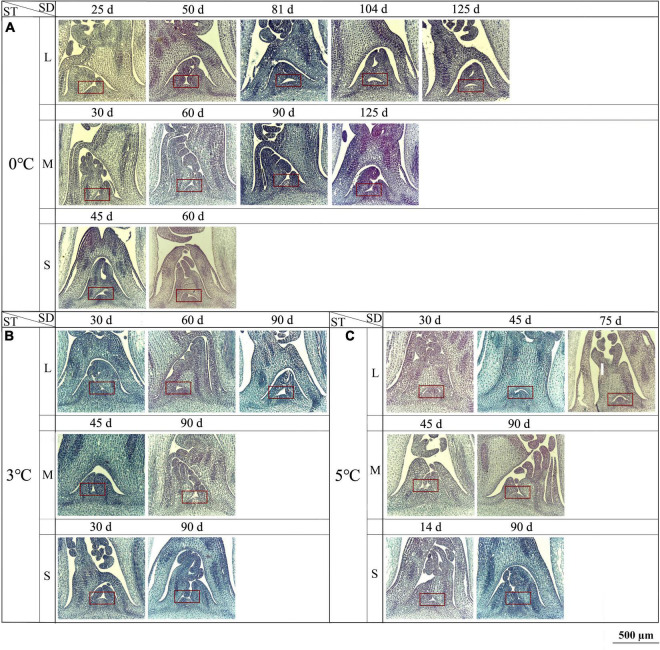
Changes in longitudinal anatomical structure (10× magnification) of the SAM seedlings of *Angelica sinensis* at different storage temperatures and durations. The red frame shows the SAM. Panels **(A–C)** represent the structure of the SAM at 0, 3, and 5°C, respectively. SD, storage duration; ST, storage temperature; L, large size; M, medium size; S, small size. n = 10 per stage for the different size seedlings.

At 0, 3, and 5°C, obvious changes in the SAM were observed. At 0°C, the interval of a new LP formation was 20–30 d (i.e., 25, 50, 81, 104, and 125 d) for the large size, 30–35 d (i.e., 30, 60, 90, and 125 d) for medium size, and 45 d (i.e., 45 and 90 d) for small size (Figure 6A). At 3°C, the interval was 30 d (i.e., 30, 60, and 90 d) for the large size, 45 d (i.e., 45 and 90 d) for medium size, and 60 d (i.e., 30 and 90 d) for small size (Figure 6B). At 5°C, the interval was 30–45 d (i.e., 30, 45, and 75 d) for the large size, 45 d (i.e., 45 and 90 d) for medium size, and 76 d (i.e., 14 and 90 d) for small size (Figure 6C). At −3 and −5°C, little change was observed for the large, medium, and small sizes (Supplementary Figure 6). The specific anatomical structures of different size seedlings at different storage temperatures (0, 3, 5, −3, and −5°C) were shown in Supplementary Figure 6.

### Effect of Storage Temperature and Duration on Root Activity

**FIGURE 7:**
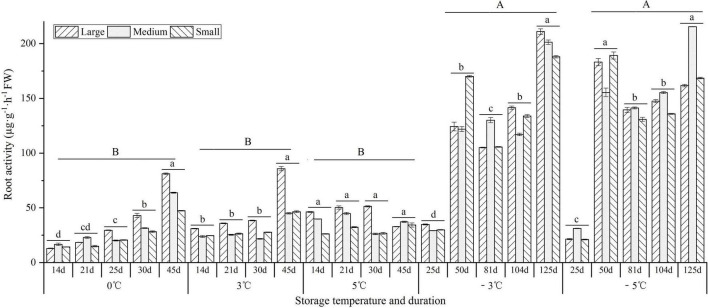
Changes in the root activity of seedlings at different storage temperatures and durations. *n* = 10 per stage for the different size seedlings. Different uppercase letters represent a significant difference (*P* < 0.05) in the average value of different seedlings and storage durations among different temperatures. Different lowercase letters represent a significant difference in the average value of different seedlings between the storage durations for the same temperature.

As shown in Figure 7, the root activity showed an increase trend with prolonged storage at 0 and 3°C, while it showed a decrease trend at the 5°C. At −3 and −5°C, the root activity maintained higher levels (over 105 μg⋅g^–1^⋅h^–1^) from 50 to 125 d than early stage 25 d (35 μg⋅g^–1^⋅h^–1^). Moreover, the root activity was significantly greater at the −3 and −5°C than that at 0, 3, and 5°C.

### Effect of Storage Temperature and Duration on Primary Metabolism

**FIGURE 8:**
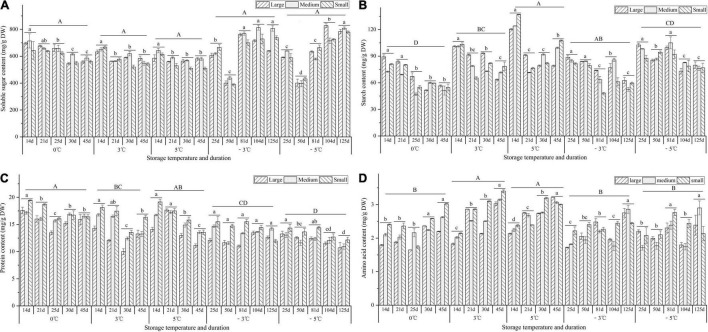
Changes in the contents of soluble sugar, starch, protein, and amino acid of seedlings at different storage temperatures and durations. Panels **(A–D)** represent the contents of soluble sugar, starch, protein, and amino acid, respectively. *n* = 10 per stage for the different size seedlings. Different uppercase letters represent a significant difference (*P* < 0.05) in the average value of different seedlings and storage durations among different temperatures. Different lowercase letters represent a significant difference in the average value of different seedlings between the storage durations for the same temperature.

For the soluble sugar contents, there was a 1.2-, 1.2-, and 1.1-fold decrease at 0, 3, and 5°C, respectively, from 14 to 45 d; there was a 1.5- and 1.5-fold decrease from 25 to 50 d, but a 1.8- and 1.9-fold increase from 50 to 125 d at the −3 and −5°C (Figure 8A). For the contents of starch and protein, decrease trends were observed at 0, 3, 5, −3, and −5°C, with 1.5-, 1.4-, 1.3-, 1.5-, and 1.2-fold decrease for starch contents (Figure 8B) and a 1.1-, 1.2-, 1.3-, 1.1-, and 1.2-fold decrease for protein contents (Figure 8C). For the amino acid contents, a 1.2-, 1.6-, 1.4-, 1.4-, and 1.2-fold increase were observed at 0, 3, 5, −3, and −5°C, respectively (Figure 8D).

### Effect of Storage Temperature and Duration on Endogenous Hormones

**FIGURE 9:**
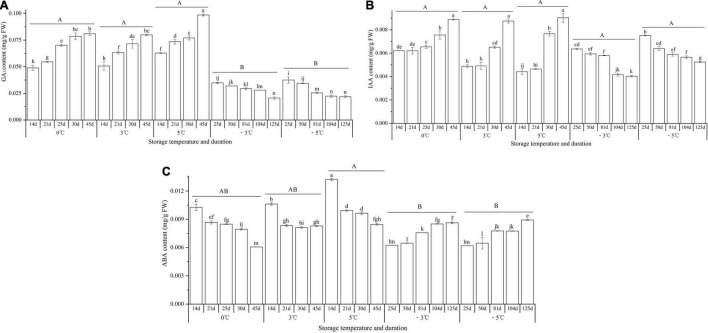
Changes in the contents of GA_3_, IAA, and ABA of seedlings at different storage temperatures and durations. Panels **(A–C)** represent the contents of GA_3_, IAA, and ABA, respectively. *n* = 10 per stage for the different size seedlings. Different uppercase letters represent a significant difference (*P* < 0.05) in the average value of different storage durations for the medium seedlings among different temperatures. Different lowercase letters represent a significant difference between the storage durations for the same temperature.

At 0, 3, and 5°C, the contents of GA_3_ and IAA exhibited significant increases from 14 to 45 d, with 1.7-, 1.6-, and 1.6-fold increase for GA_3_ (Figure 9A) and 1.4-, 1.8-, and 2.0-fold increase for IAA, respectively (Figure 9B), while at −3 and −5°C, the contents of GA_3_ and IAA exhibited significant decreases from 25 to 125 d, with 1.7- and 1.7-fold decrease for GA_3_ (Figure 9A) and 1.6- and 1.4-fold decrease for IAA, respectively (Figure 9B); for the ABA contents, significant decreases were observed at 0, 3, and 5°C, with 1.7-, 1.3-, and 1.6-fold decrease from 14 to 45 d; significant increases were observed at −3 and −5°C, with 1.4- and 1.4-fold increase from 25 to 125 d, respectively (Figure 9C).

## Discussion

Vernalization plays a crucial role in promoting plant flowering by prolonged exposure to low temperature (Amasino, 2005). Plants differ in the species and age at which they become sensitive to vernalization; thus, there are great differences in the cold requirement for flowering as well as anatomical structures and metabolites in the SAM; in addition, the vernalization can be lost at high temperature or avoided at freezing temperatures (Taiz and Zeiger, 2010b). For *A. sinensis*, the precise temperature and duration to complete the vernalization of seedlings have not been revealed, although several investigations strived to uncover the effective temperatures range for vernalization and find out the changes in metabolites (Wang, 1977; Li, 1979; Zhang and Huang, 1998; Chen et al., 2014; Jia et al., 2018). Here, we have found that vernalization temperature (VT) and duration (VD) are affected by the seedling sizes, the VD of seedlings ranges from 57 to 85 d with VT from 0 to 5^°^C, the early bolting and flowering (EBF) is effectively inhibited by seedlings were stored at the −3 to −5^°^C, and physiological characteristics significantly altered at different storages.

Extensive experiments have proved that temperature affects the quality of fruits, seeds, and seedlings during postharvest storage (Wilson et al., 2000; Cuadra-Crespo and Amor, 2010; Thomas et al., 2017). Rotting or chilling damages will occur when the tissues or organs are stored at non-freezing or freezing temperatures for a long time (Taiz and Zeiger, 2010a; Watkins, 2017). Previous studies have demonstrated that the seedlings of *A. sinensis* could germinate and grow after non-freezing (0 to 5°C) and freezing temperatures (−2 to −13°C) storage over 150 d (Wang, 1977, 1979; Wang and Zhang, 1982; Jia et al., 2018). In this study, the RR of seedlings increased with temperatures elevated from 0 to 5°C (*ca.* 49% at 5°C for 45 d), the FIR increased with temperatures declined from 0 to −5°C (*ca.* 8% at −5°C for 125 d) (see Figure 1), and the GR increased with temperatures declined from 5 to −5°C (*ca.* 94% at −5°C for 125 d) (see Figure 2).

The developmental signals that transit from vegetative growth to flowering include endogenous factors (e.g., circadian rhythms, phase change, and hormones) and external factors (e.g., photoperiodism and vernalization) (Taiz and Zeiger, 2010b). Furthermore, there is a linear relationship of apex diameter (AD) of *Brassica oleracea* with VT and VD, with the model *dAD*/*dVD* = *f*[*VT*(*VD*)] (Wurr et al., 1993). For the *A. sinensis*, a positive relationship of the EBF rate with the size seedlings has been observed (Lin et al., 2007; Jia et al., 2018). The effective VT for seedlings ranged from 0 to 10°C, with an optimum range between 0 and 5°C (Wang, 1977, 1979; Jia et al., 2018). In this study, there was a linear relationship of EBF rate with VT, VD, and SC, and the precise VD was predicted to range from 57 to 85 d for different size seedlings at the VT from 0 to 5^°^C (see Figure 4B). Interestingly, the 52.2% EBF rate for the seedlings stored at 3°C for 45 d in this controlled experiment (see Figure 3) was almost consistent with *ca.* 40% EBF rate at 2.1°C for 33 d and 3.2°C for 44 d in the practical overwintered storage (see Supplementary Figure 5 and Supplementary Table 3). In addition, several investigations have found that the EBF is also affected by the environmental factors (e.g., drought, latitude, and altitude), soil nutrients (e.g., nitrogen and phosphorus fertilizers), and planting density (Wang, 1977; Qi et al., 2004; Qiu et al., 2010).

Vernalization can be lost as a result of exposure to de-vernalizing conditions (e.g., high temperature) or freezing temperatures at which metabolic activity is suppressed (Taiz and Zeiger, 2010b). Previous studies on *A. sinensis* have found that the vernalization can be largely lost when seedlings exposure to 33–35°C for 2 d with the EBF rate of 20% or significantly avoided when seedlings exposure to −2 to −12°C for 210 d with the EBF rates 5.6–0% (Wang, 1979; Wang and Zhang, 1982). In this study, the vernalization can be avoided when the seedlings were stored at −3 to −5°C for 125 d, with no EBF occurrence (see Figure 3).

During the vernalization, stable changes (e.g., cell division, DNA replication, and gene expression) in the SAM are required (Taiz and Zeiger, 2010b; Dai et al., 2021). The SAM is a dynamic structure that changes during its cycle of leaf and stem formation, and the transition from vegetative to reproductive development is marked by an increase in the frequency of cell divisions within the central zone (Taiz and Zeiger, 2010b). Previous literature has reported that the number of *A. sinensis* leaves in one reproductive cycle always ranges from 12 to 14, and a physiological age developing into flowering would be advanced with one more new leaf producing (Tang, 1980). In this study, there were regular structural changes in the SAM for the different seedlings exposure to different temperatures and durations, with more leaves producing at the VT (0, 3, and 5°C), while little change at freezing temperatures (−3 and −5°C) (see Figure 6), which shows that the vernalization accelerates the leaves formation and eventually the physiological age. Similar results of morphological changes in the SAM have also been observed in other plants in response to vernalization, such as *Lolium temulentum*, *Antirrhinum majus*, *Brunonia australis*, and *Calandrinia* (Arumuganathan et al., 1991; Adams et al., 2003; Cave et al., 2011). The acceleration of growth and development can be induced from the differential expression of genes, such as *SUPPRESSOR OF OVEREXPRESSION OF CONSTANS 1* (*SOC1*), *AGAMOUS-LIKE19* (*AGL19*), and *FLOWERING LOCUS C* (*FLC*) (Sheldon et al., 2000; Taiz and Zeiger, 2010b; Suter et al., 2014; Jaudal et al., 2018).

During the vernalization, active metabolism in the SAM is also required (Taiz and Zeiger, 2010b). Previous studies on *A. sinensis* found that the soluble sugar content decreased, but the nitrate reductase activity increased after the seedlings exposure to 4°C for 170 d (Zhang and Huang, 1998); the contents of soluble sugar and protein decreased, but the malonic dialdehyde content increased after the seedlings exposure to −10°C for 210 d (Chen et al., 2014). In this study, the levels of soluble sugar, starch, protein, and ABA in the seedlings decreased, but the levels of root activity, amino acid, GA_3_, and IAA exhibited an increase trend after the seedlings were stored at the VT (0, 3, and 5°C) for 45 d; the levels of root activity, soluble sugar, amino acid, and ABA increased, but the levels of starch, protein, GA_3_, and IAA decreased after the seedlings were stored at freezing temperatures (−3 and −5°C) for 125 d (see Figures 7–9). These changes in physiological characteristics can well explain the previously measured index, for example, the root activity is in accordance with the germination rate, the levels of GA_3_ and IAA involved in growth and development are in accordance with the EBF rate, and the levels of soluble sugar, amino acid, and ABA involved in stress tolerance are in accordance with the freezing injury rate. Similar results of physiological changes in the seedlings have also been observed in other plants in response to vernalization and freezing temperatures, such as a decrease in starch and total nitrogen in cabbage after vernalization (Zhao et al., 2010), a general increase in neutral and acidic amino acids in both spring and winter wheat varieties grown at 2°C for 2 weeks (Trione et al., 1967), a significant increase in GA and related biosynthetic genes (*ent-kaurene oxidase* and *GA20-oxidase*) in Pak Choi after 4°C treatment (Shang et al., 2017), an increase in IAA and related biosynthetic genes (*IAA8* and *nitrilase/nitrile hydratase NIT4*), and a decrease in ABA and related biosynthetic genes (*ABA2* and *9-cis-epoxycarotenoid dioxygenase NCED1*) in *Beta vulgaris* after 4°C treatment for 16 weeks (Liang et al., 2018).

## Conclusion

From the above observations, the vernalization of *A. sinensis* seedlings is dynamically regulated by temperature and duration, and the EBF rate can be effectively inhibited with the seedlings stored below freezing temperatures. Based on the linearization regression analysis, a model of the EBF rate with vernalization temperature and vernalization duration has been proposed for different size seedlings. The epigenetic regulation mechanism of vernalization will be examined *via* future transcriptomics and epigenomics investigations.

## Data Availability Statement

The original contributions presented in the study are included in the article/Supplementary Material, further inquiries can be directed to the corresponding authors.

## Author Contributions

XL and MML: investigation, data curation, and writing—original draft. MFL: conceptualization, writing—review and editing, and project administration. JW: conceptualization and project administration. All authors contributed to the article and approved the submitted version.

## Conflict of Interest

The authors declare that the research was conducted in the absence of any commercial or financial relationships that could be construed as a potential conflict of interest.

## Publisher’s Note

All claims expressed in this article are solely those of the authors and do not necessarily represent those of their affiliated organizations, or those of the publisher, the editors and the reviewers. Any product that may be evaluated in this article, or claim that may be made by its manufacturer, is not guaranteed or endorsed by the publisher.

## Abbreviations

EBF: early bolting and flowering
FIR: freezing injury rate
FT: freezing temperature
GR: germination rate
RR: rotting rate
SAM: shoot apical meristem
SC: seedling classification
VD: vernalization duration
VT: vernalization temperature.
