# Supplementary material for: Depicting Precise Temperature and Duration of Vernalization and Inhibiting Early Bolting and Flowering of Angelica sinensis by Freezing Storage
Source: Front Plant Sci. 2022 May 19;13:853444. doi: 10.3389/fpls.2022.853444 (PMC9161173; doi:10.3389/fpls.2022.853444)
Supplement: Supplementary file 1 [file Data_Sheet_1.docx]

**Supplemental materials**

**Figure supplemental materials**


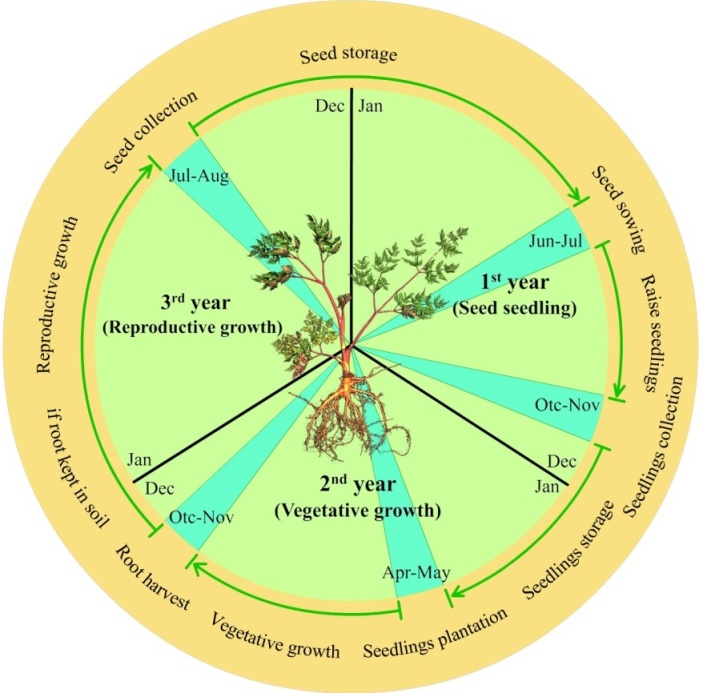


**Fig. S1.** Process of growth and development of *Angelica sinensis*.

**
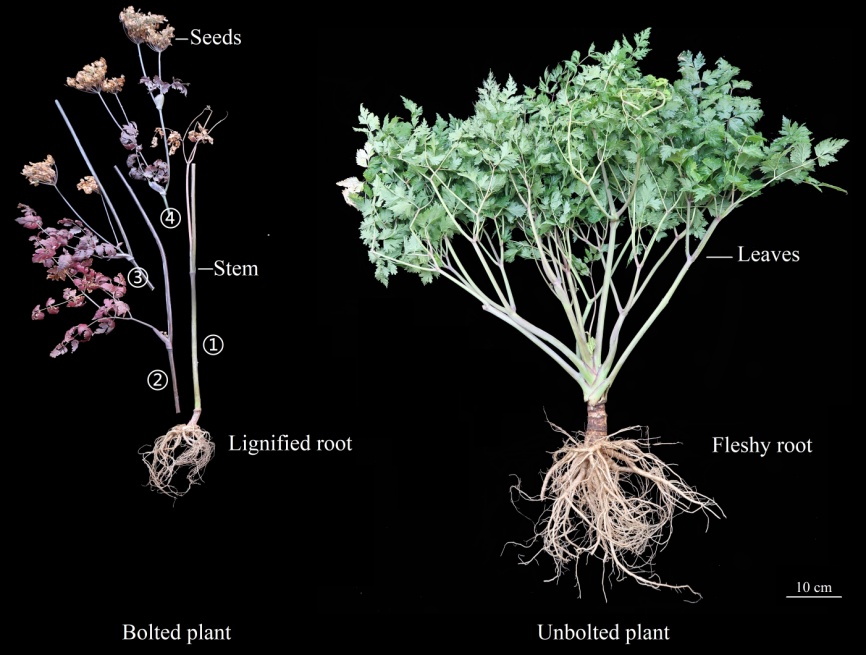
**

**Fig. S2.** Morphological characteristics of lignified (bolted) and fleshy (unbolted) of *Angelica sinensis*. The ①, ②, ③ and ④ in the image refer to the order of organs that are cut and separated from root to aerial parts.

**
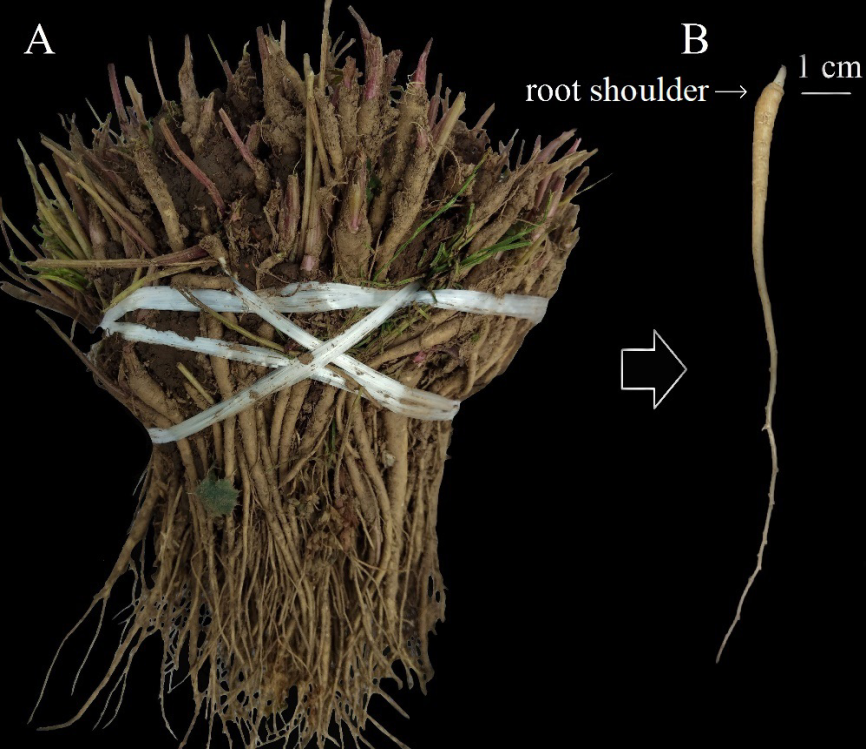
**

**Fig. S3**. The tied seedlings (A) and single seedling (B) of *Angelica sinensis*.


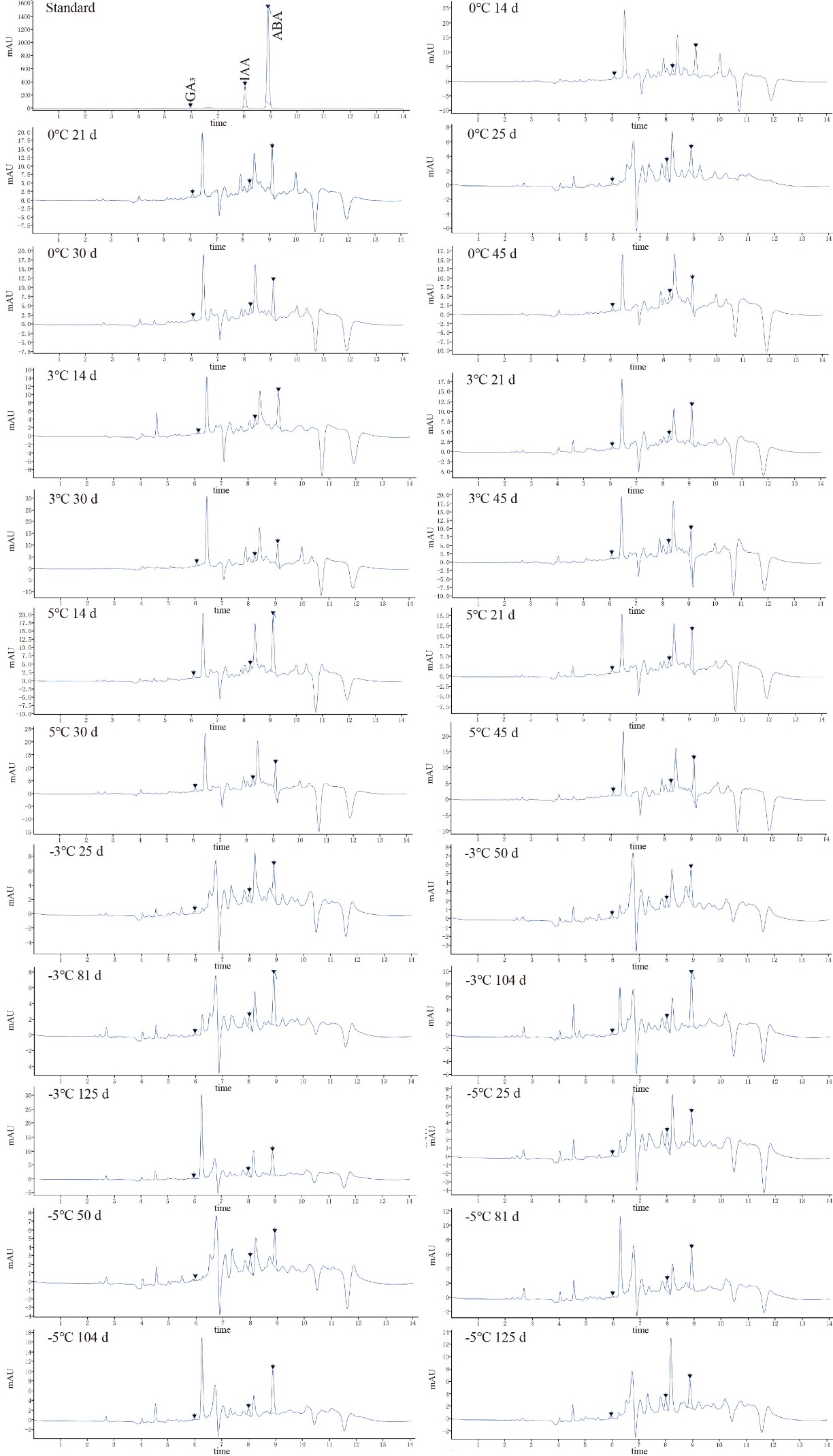


**Fig. S4.** Representative chromatograms of standard references and samples at different temperatures and durations.

**
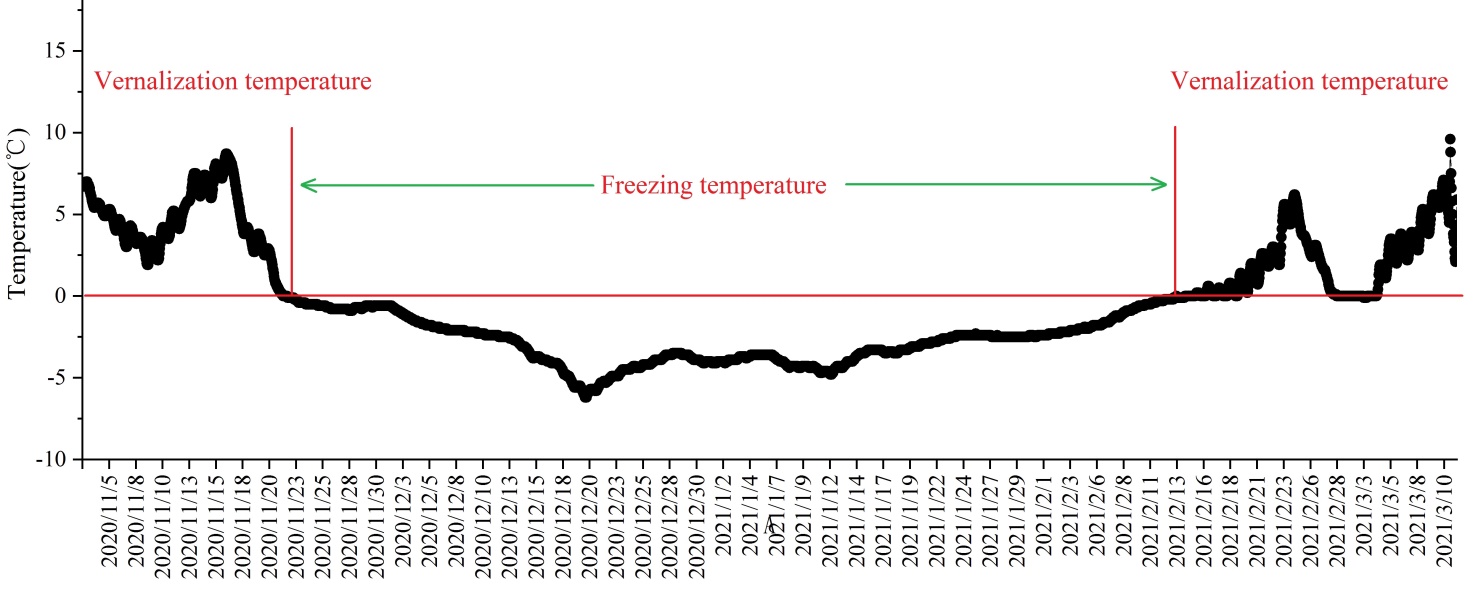
**

**Fig. S5.** Changes in temperature during the practical overwinter storage.


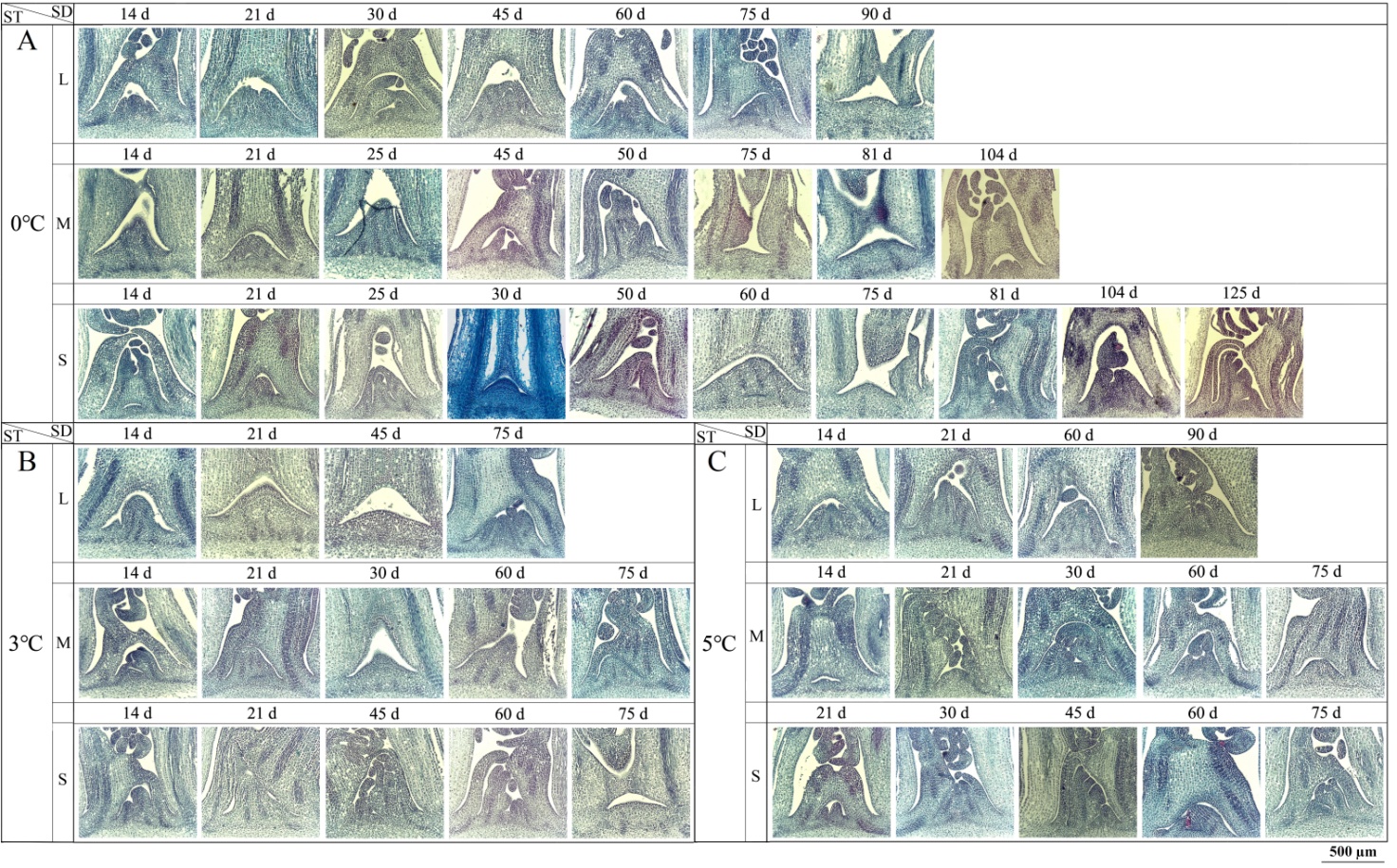


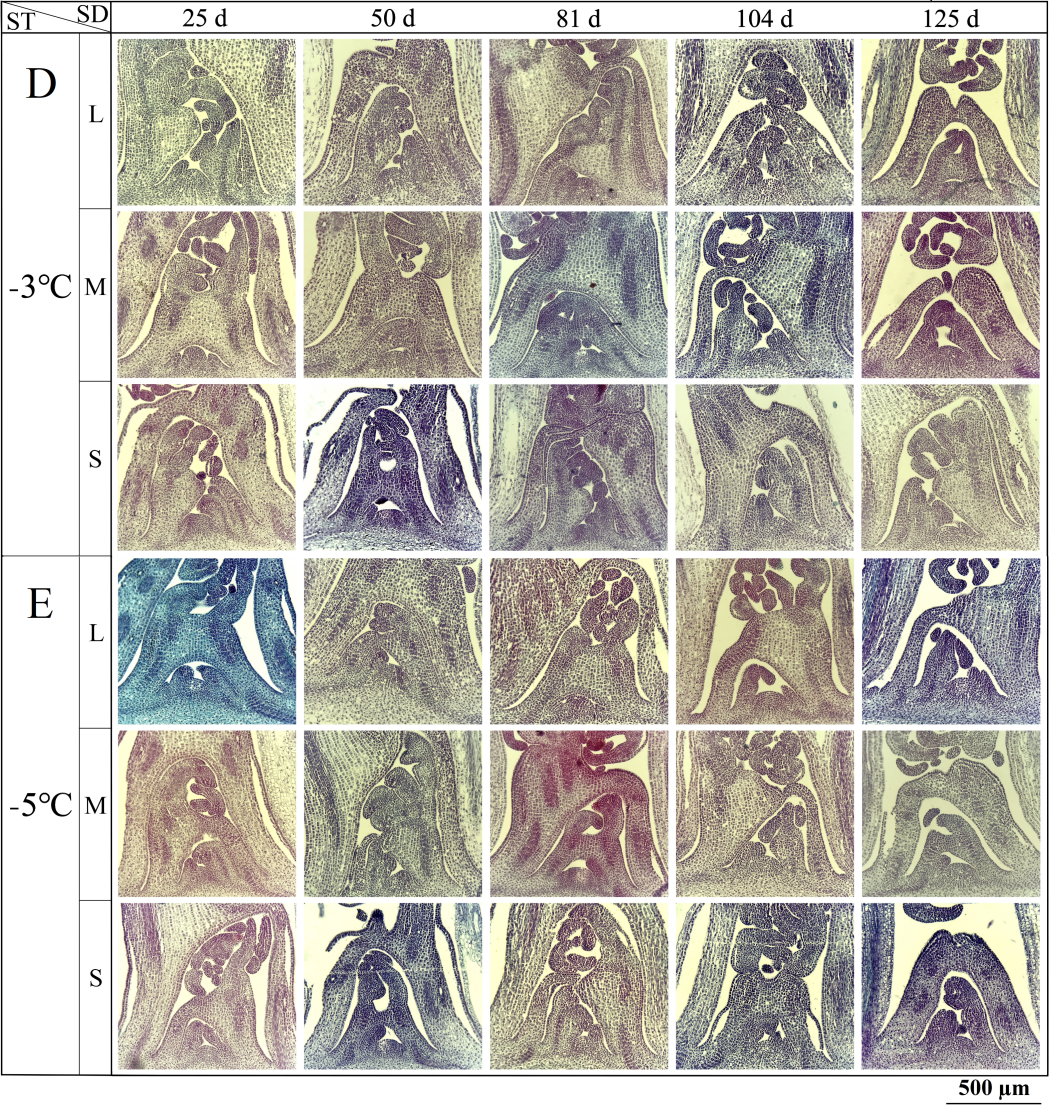


**Fig. S6.** Specific anatomical structures of different size seedlings at different storage temperatures.

**Table supplemental legends**

**Table S1.** Standard curves of TTC, soluble sugar, starch, protein and amino acid.

| **Reference** | **Standard curve** | ***R^2^*** |
| --- | --- | --- |
| TTC | *C* = 23.32*A* + 0.84 | 0.9997 |
| Soluble sugar | *C* = 72.99*A* - 1.47 | 0.9968 |
| Starch | *C* = 238.10*A* - 9.60 | 0.9914 |
| Protein | *C* = 142.86*A* - 12.60 | 0.9951 |
| Amino acid | *C* = 71.94*A* + 0.54 | 0.9986 |

“*C*” represents the compound amount (µg), and “*A*” represents the absorbance.

**Table S2.** Standard curves of GA_3_, IAA and ABA.

| **Reference** | **Standard curve** | ***R^2^*** |
| --- | --- | --- |
| GA_3_ (B20187) | *X*=6.7431*Y*+1.8166 | 0.9993 |
| IAA (B21810) | *X*=0.3019*Y*-0.2167 | 0.9996 |
| ABA (B27484) | *X*=0.0448*Y*+1.5521 | 0.9988 |

“*X*” represents the compound concentration (µg/mL), and “*Y*” represents the peak area (AU). The agents GA3, IAA and ABA were purchased from Shanghai yuanye Bio-Technology Co., Ltd, China.

**Table S3.** The data of storage temperatures and durations as well as EBF rate in the practical large-scale cultivation.

| **Actual temperature (**°C**)** | **Average temperature (**°C**)** | **Duration days (d)** | **EBF rates (%)** |
| --- | --- | --- | --- |
| 0-5 | 2.13 | 33 | >40 |
| 0-10 | 3.22 | 44 | >40 |
